# Sex differences in the blood–brain barrier and neurodegenerative diseases

**DOI:** 10.1063/5.0035610

**Published:** 2021-03-16

**Authors:** Callie M. Weber, Alisa Morss Clyne

**Affiliations:** Fischell Department of Bioengineering, University of Maryland, College Park, Maryland 20742, USA

## Abstract

The number of people diagnosed with neurodegenerative diseases is on the rise. Many of these diseases, including Alzheimer's disease, Parkinson's disease, multiple sclerosis, and motor neuron disease, demonstrate clear sexual dimorphisms. While sex as a biological variable must now be included in animal studies, sex is rarely included in *in vitro* models of human neurodegenerative disease. In this Review, we describe these sex-related differences in neurodegenerative diseases and the blood–brain barrier (BBB), whose dysfunction is linked to neurodegenerative disease development and progression. We explain potential mechanisms by which sex and sex hormones affect BBB integrity. Finally, we summarize current *in vitro* BBB bioengineered models and highlight their potential to study sex differences in BBB integrity and neurodegenerative disease.

## I. INTRODUCTION

**FIG. 1.**
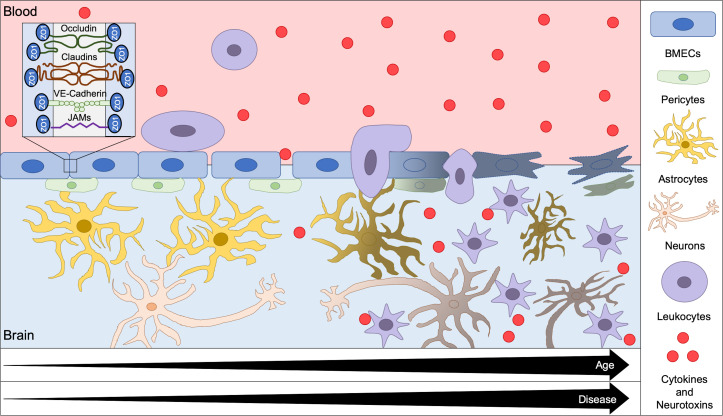
Overview of BBB degradation with age and disease. The BBB, which is formed by BMECs and maintained through interactions with pericytes and astrocytes, restricts cell and molecule movement from the blood into the brain. BMECs form impermeable intercellular junctions through tight junction proteins, including occludins, claudins, VE-Cadherin, JAMs, and ZO proteins. With the increasing age and disease, tight junction proteins degrade, leading to BBB opening. The leaky BBB allows cytokines, neurotoxins, and leukocytes to infiltrate the brain, which can cause downstream inflammation and neurodegeneration.

The brain has an extensive microvascular network since metabolic demand requires brain cells not more than 200 *μ*m from a capillary to survive.^1^ The blood–brain barrier (BBB) tightly controls nutrient and waste product exchange between the blood and all cell types in the brain. The BBB is formed primarily of brain microvascular endothelial cells (BMECs), which are connected via tight junction proteins including claudins, occludins, junction adhesion molecules (JAMs), and zonula occludins (ZO) (Fig. 1). Claudin family proteins are considered the primary BBB sealing component, with claudin-5 having the greatest BBB expression compared to other isoforms.^2,3^ Occludins, the first tight junction proteins to be discovered, regulate adhesion properties between BMECs.^4^ JAMs, and specifically JAM-1, are essential for tight junction initiation between BMECs.^5^ JAM down-regulation or deletion markedly increased BBB permeability.^5,6^ ZO-1, the primary ZO protein expressed in the BBB, is a cytoplasmic scaffolding protein that tethers transmembrane tight junction proteins to the actin cytoskeleton.^7^ Tight junction assembly and function are also influenced by signaling with other adhesion complexes, including adherens junctions and focal adhesions.^8^

The BBB is further supported by pericytes and astrocytes. Pericytes are mural cells that surround the brain microvasculature where they stabilize the vascular wall. Pericytes maintain the BBB by releasing signaling factors that impact BMEC tight junction proteins,^9^ secreting basement membrane proteins,^10^ regulating neuroinflammation,^11^ and contracting via proteins such as α-smooth muscle actin to control blood flow in the brain.^12^ Astrocyte terminal processes (endfeet) also directly contact BMECs to contribute to BBB strength. Astrocytes secrete factors, including growth factors, which regulate BMEC tight junction formation^13^ and transporter expression and polarization.^14,15^ Together, BMECs, pericytes, and astrocytes maintain strict control of molecule movement across the BBB, which is essential for protecting the brain from neurotoxins.

The BBB degrades with the increasing age, which promotes inflammation and neurotoxicity. BBB dysregulation is further observed in neurodegenerative diseases such as Alzheimer's disease (AD), Parkinson's disease (PD), multiple sclerosis (MS), and motor neuron diseases (MNDs) (Fig. 1). Although it is debated whether BBB breakdown is causal to or a by-product of neurodegeneration, it is clear that increased BBB permeability leads to neurotoxin and leukocyte infiltration into the brain, initiating an immune response and propagating cell death. Zhao *et al.* proposed a model of vascular-mediated neurodegeneration in which atypical intercellular BBB signaling results in increased BBB permeability, which, in turn, leads to brain accumulation of (1) hemoglobin and iron, which increase oxidative stress, (2) fibrinogen, thrombin, and plasminogen, which degrade the brain extracellular matrix and activate microglia, and (3) albumin, which leads to cerebral hypoperfusion, hypoxia, and edema.^16^ Together, these BBB degradation effects contribute to the neuronal stress and eventual cell death associated with neurodegenerative diseases; however, the events that incite BBB dysfunction have yet to be elucidated.

Sexual dimorphisms are abundant in neurodegenerative diseases *in vivo*,^17^ and *in vivo* studies similarly suggest that sex hormones play a role in BBB integrity.^18^ Indeed, estrogen treatment protects the brain from the immune response under inflammatory conditions by enhancing BBB functionality and decreasing leukocyte extravasation across the BBB^19^. The overall neuroprotective effects of estrogen are further described in a number of review articles, including those by Brann *et al.*,^20^ Raghava *et al.*,^21^ and Zárate *et al.*^22^ Hormone-dependent and independent sex differences in endothelial function are also closely linked with sexual dimorphisms in cardiovascular disease;^23^ however, sex differences in the BBB and their role in neurodegenerative diseases are less understood.

In this Review, we describe sex differences in neurodegenerative diseases and the BBB, potential mechanisms by which sex impacts the BBB, and current BBB bioengineered *in vitro* models. We conclude by proposing ways in which cell sex, hormone exposure, and other sex-related differences can be incorporated into studies of how sex alters the BBB and subsequent neurodegenerative diseases.

## II. SEX DIFFERENCES IN NEURODEGENERATIVE AND NEUROLOGICAL DISEASES

As of 2020, an estimated 5.8 million Americans are diagnosed with Alzheimer's disease (AD).^24^ An additional 1 million Americans are diagnosed with Parkinson's disease (PD),^25^ 914 000 with multiple sclerosis (MS),^26^ and 63 000 with motor neuron disease (MND).^27^ While these are some of the most prevalent neurodegenerative disorders, the National Institute of Neurological Disorders and Stroke (NINDS) reports over 600 neurological disorders that affect an estimated 50 million Americans.^28^ BBB breakdown is a common denominator in many neurodegenerative and neurological diseases.^29,30^ Whether this breakdown is a cause or an effect of neurodegeneration is yet to be elucidated; however, based on disease prevalence in male vs female patients, sex likely plays a role in the BBB disruption associated with these diseases.

### A. Alzheimer's disease

Alzheimer's disease (AD), first reported in 1907, is marked by progressive cognitive impairment.^31^ There are a number of hypotheses for AD onset, as reviewed by Liu *et al.,* including the neurovascular hypothesis. In this hypothesis, BBB dysregulation leads to neurovascular uncoupling, followed by cerebral hypoperfusion, hypoxia, and inflammation.^32^ These processes could then produce the mild cognitive impairment associated with AD onset and propagate over time, leading to a further cognitive decline.

Almost two thirds of Americans diagnosed with AD are women,^24^ which could be caused either by increased AD incidence in women vs men or because women have a longer lifespan than men.^33^ Recent studies showed that female subjects with the apolipoprotein ε4 (APOE4) allele, which increases AD risk by almost 15%,^34^ are more likely to be diagnosed with AD than male subjects with the gene.^35^ Additional AD risk factors also have sex-specific effects. For example, obesity leads to greater BBB disruption and induces a larger inflammatory response in women than men.^36,37^

Many AD sex differences can be linked to the neuroprotective effects of estrogen. *In vitro* studies demonstrate that estrogen regulates BBB glucose transporter expression and membrane translocation,^38,39^ which could impact glucose transport into the brain. Indeed, in human studies, brain glucose transport, and thus brain glucose metabolism, decreased following the menopausal transition.^40,41^ Similarly, preclinical studies indicate that estrogen decreases brain reactive oxygen species (ROS) production,^42^ which is linked to decreased tight junction protein expression and BBB dysregulation.^43^ Unfortunately, clinical trials have shown little to no effect of estrogen replacement on AD.^44^ However, a recent proteomic analysis of estrogen-impacted pathways suggests that previous clinical trials targeted estrogen receptors (ERs) too late and for too short of a time, whereas early and long-term estrogen treatment may reduce AD pathogenesis.^45^

### B. Parkinson's disease

Parkinson's disease (PD) is a neurodegenerative disease diagnosed by bradykinesia and tremor or muscle rigidity. PD results from dopaminergic neuron loss in the substantia nigra and is marked by cytoplasmic aggregates, called Lewy bodies, in the remaining neurons.^46^ Potential PD pathogenesis mechanisms include the theory of neuroinflammation, which suggests that alpha synuclein, the primary structural Lewy body component, triggers macrophage activation that contributes to dopaminergic neuron degeneration.^46^

Men are 1.5 times more likely to be diagnosed with PD than women.^47^ Neuroprotective estrogen effects may further cause women to have a benign presentation in the preclinical PD phase. This benign presentation may contribute to PD being diagnosed 2.1 years later in female patients than in male patients.^48^ Although estrogen appears to delay PD onset, once symptoms develop, there are no clear differences in PD progression between men and women.^48^ A review by Miller *et al.* emphasizes that research on sex disparities in PD development and symptomology, as well as the neuroprotective role of estrogen, has led to murky conclusions.^49^ Thus, additional research in sex differences in PD is essential for understanding and preventing disease onset.

The role of the BBB in PD remains uncertain. Initial studies on blood-cerebral spinal fluid (CSF) barrier integrity in early PD indicated that this barrier did not demonstrate dysfunction or contribute to PD development.^50^ More recently, a study of PD patients demonstrated increased vascular endothelial growth factor (VEGF) and other angiogenic markers compared to controls, which correlated with increased BBB permeability measured using the CSF/plasma albumin ratio.^51^ Additionally, increased reactive microglia in PD patients^52^ and increased concentrations of inflammatory cytokines, such as interleukin 6 (IL-6) and interleukin 1 beta (IL1-β) in CSF from PD patients,^53^ could be linked to degraded BBB tight junction proteins and increased BBB permeability.^54^ Mouse models also suggest the PD-associated increase in the leukocyte adhesion molecule intercellular adhesion molecule 1 (ICAM-1) on the brain endothelium,^55^ which may contribute to downstream macrophage activation and dopaminergic neuron degeneration. VEGF-mediated angiogenesis is increased after brain estrogen receptor activation, and ICAM-1 expression is regulated by estradiol, the most prevalent estrogen form.^56,57^ Thus, estrogen may contribute to some of the sexual dimorphisms associated with PD.

### C. Multiple sclerosis

Multiple sclerosis is an inflammatory disease that results in neuron demyelination and symptoms such as sensory deficits, fatigue, and muscle weakness.^58^ Although MS is primarily thought of as an autoimmune disorder, cerebrovascular dysregulation may lead to leukocyte transmigration across the BBB, triggering the MS inflammatory cascade. Furthermore, cerebrovascular inflammation as a result of the MS autoimmune response leads to BBB breakdown, facilitating transendothelial leukocyte migration and increasing axon demyelination and MS symptoms.^59^

Women are diagnosed with MS more frequently than men.^60^ However, variation in the regional female to male ratio of MS diagnosis indicates interactions between sex and genetic, environmental, and cultural differences. While Sweden reports a female to male ratio of 2.35:1,^61^ Canada reports a ratio of 2.17:1, with prevalence varying among immigrants of different heritage.^62^ Voskuhl and Gold reviewed potential mechanisms through which men may be less susceptible to MS, including protective effects of testosterone, Y chromosome genes that reduce susceptibility, and lifestyle and environmental differences.^63^

Research on MS sexual dimorphisms using the experimental autoimmune encephalomyelitis (EAE) Swiss Jim Lambert (SJL) mouse model elucidated a link between sex and BBB degradation. Sphingosine-1-phosphate receptor 2 (S1PR2) is upregulated in BMECs of female MS mouse models compared to male MS mouse models or healthy controls. S1PR2 activates the RhoA/Rho-associated protein kinase (ROCK) pathway, which pulls apart BBB tight junction proteins.^64^ Additional research is needed to validate S1PR2 as a sex-specific mechanism by which MS disproportionately affects women.

### D. Motor neuron disease

In 2016, 330 918 people worldwide were living with a diagnosed motor neuron disease (MND), including amyotrophic lateral sclerosis (ALS), spinal muscular atrophy, hereditary spastic paraplegia, primary lateral sclerosis, progressive muscular atrophy, and pseudobulbar palsy.^27^ MND leads to degeneration of upper and lower motor neurons, causing progressive weakness and eventual respiratory failure.^65^ MND biomarkers include increased serum matrix metalloproteinases,^66^ immunoglobulin G (IgG), and immune complexes (ICs),^67^ which are all associated with BBB breakdown.

The lifetime risk of MND diagnosis is 1 in 472 women and 1 in 350 men, indicating a 54% higher MND diagnosis likelihood in men than in women.^68^ A meta-analysis of MND patients in France demonstrated a lower incidence of MND diagnosis in women compared to men at all ages; however, this difference is more pronounced in young people (20–49 years) and becomes less prominent with age. These data suggest that around the menopausal transition, women become more susceptible to MNDs.^69^ Ovariectomized female mouse ALS models demonstrated accelerated disease progression similar to male mice, while estradiol treatment slowed disease progression.^70^ Thus, female sex hormones may be protective against MND progression.

While the MND manifestation differs based on specific diagnosis, BBB disruption is thought to be a common thread among these diseases. MNDs, and ALS in particular, are associated with increased ROS, which activate myosin light chain (MLC) kinase and, thus, pull apart BBB tight junctions.^71^ Estrogen downregulation of phosphorylated MLC is one mechanism through which female sex may be protective against BBB degeneration and MND development.^72^ Mechanisms by which sex may disproportionately affect MND development are understudied, and while Kakaroubas *et al.* propose disproportionate telomere shortening, circadian rhythms, and oxidative stress to be BBB disruptors associated with MND,^71^ the role of sex in these processes is yet to be elucidated.

### E. Other neurological disorders

Women have a higher lifetime risk of stroke than men due to their longer life expectancy and the increasing stroke risk with age.^73^ *In vitro* studies demonstrated that treating mouse brain endothelial cells with estradiol for 24–48 h prevented oxygen-glucose deprivation (OGD)-induced cell death, suggesting that sex hormones may protect against ischemic-stroke in pre-menopausal women.^74^ Additionally, neurons from men were more susceptible to nitrosative stress than neurons from women,^75^ and astrocytes from neonatal female rat brains were more resilient to OGD than male astrocytes.^76^ Thus, several cells of the female BBB may be less vulnerable to and less impacted by ischemic stroke than cells of the male BBB.

Glioblastoma (GBM) is the most aggressive form of brain cancer and is 1.57 times more likely to occur in men than in women.^77^ Male sex may also be associated with shorter survival. Interestingly, temozolomide, a common chemotherapeutic used to treat GBM, is more efficacious in women than in men.^78^ While there are no currently known links between sex-dependent GBM incidence or drug efficacy and BBB sexual dimorphisms, *de novo* GBM vascularization contributes to variable BBB properties throughout the tumor. GBM has tumor regions of intact BBB, which reduces drug delivery to the tumor, as well as tumor regions with a disrupted BBB.^79^ The disrupted BBB regions are hypothesized to relate to improper astroglial polarity,^80^ disruptive soluble factor release from glioma cells,^81^ or a combination of these.

## III. SEX DIFFERENCES IN THE BBB

### A. Sex differences in BBB strength

Paracellular and transcellular transport across the BBB is strictly regulated by BMECs and the tight junctions formed between them. Low BBB permeability is consistent with an intact barrier that promotes neurological health. *In vitro* BBB paracellular permeability can be assayed using transendothelial electrical resistance (TEER), which quantifies the resistance to ion flow across the barrier.^82^ Tracer flux (e.g., fluorescent dextran of varying molecular weights) across the BBB can further be used to calculate permeability coefficients and estimate pore sizes between tight junctions.^82^

Although no *in vitro* studies specifically focused on the effect of biological sex on BBB integrity, several studies differentiated induced pluripotent stem cell (iPSC) lines from both male and female subjects into BMECs and measured their TEER values (Table I). These published data suggest that iPSC-derived BMECs from pre-menopausal women have decreased permeability, and thus increased barrier strength, compared to iPSC-derived BMECs from men. In the first reported iPSC-BMEC differentiation, the barrier strength of female IMR90–4^83^ cells and male DF19–9-11T^84^ cells in co-culture with primary rat astrocytes was compared. The male DF19–9-11T cell line demonstrated a markedly lower TEER value compared to the female IMR90–4 cell line (777 ± 112 vs 1450 ± 140).^85^ However, male DF19–9-11T cells had higher platelet endothelial cell adhesion molecule-1 (PECAM-1 or CD31) expression than the female IMR90–4 cells (75% compared to 68%).^85^ PECAM-1 is expressed in and thought to be involved in BMEC tight junction integrity,^86^ which indicates that an increase in other tight junction proteins likely leads to the greater TEER values in the female IMR90–4 BMEC.

**TABLE I.** TEER values vary between iPSC-BMECs of differing sex.

Research group	Cell kine	Sex	Age	Cell source	TEER
Lippmann *et al.*^85^^,^^a)^	DF19-9-11T	Male	Newborn	Foreskin fibroblasts	777 ± 112 Ω cm^2^
	IMR90-4	Female	Fetal	Lung fibroblasts	1450 ± 140 Ω cm^2^
Hollmann *et al.*^89^^,^^b)^	SM14	Male	40	Epidermal fibroblasts	Lowest
	CD12	Male	Newborn	Dermal fibroblasts	Middle
	CC3	Female	18	Dermal fibroblasts	Middle
	IMR90-4	Female	Fetal	Lung fibroblasts	Highest
Qian *et al.*^90^	DF19-9-11T	Male	Newborn	Foreskin fibroblasts	3571 ± 448 Ω cm^2^
	IMR90-4	Female	Fetal	Lung fibroblasts	3315 ± 702 Ω cm^2^
Grifno *et al.*^91^	BC1	Male	46	Bone marrow	4118 ± 119 Ω cm^2^
	C12	Male	Newborn	Dermal fibroblasts	1897 ± 76 Ω cm^2^

^a)^ Lippmann *et al.* reported that TEER values are in co-culture with primary rat astrocytes, while all other reported TEER values are from BMEC mono-cultures.

^b)^ Results in the study by Hollmann *et al.* are shown as graphs rather than numerical values and, therefore, are described qualitatively.

In later studies, graphs published by Hollmann *et al.* show higher TEER values for BMECs in mono-culture derived from female IMR90–4 and CC3^87^ iPSC-BMECs compared to male CD12^87^ and SM14^88^ iPSC-BMECs.^89^ However, these data disagree with TEER values measured by Qian *et al.* In their hands, male DF19–9-11T^84^ iPSC-BMECs in mono-culture had TEER values that were statistically similar to the female IMR90–4 line (3571 ± 448 and 3315 ± 702, respectively).^90^ The accuracy of this comparison may be limited by the fact that this study had 26 biological replicates for the IMR90–4 iPSC-BMEC, yet only three biological replicates for the DF19–9-11T iPSC-BMEC.^90^

While these data suggest that there may be sex-related differences in BBB barrier strength, other notable differences among the cell lines likely also contribute to the variation in TEER values. For example, Grifno *et al.* used all male iPSC lines, yet still measured large differences in TEER values. TEER variation may relate to differences in the donor age, cell tissue source, and differentiation or culture conditions in addition to sex.^91^ Thus, *in vitro* studies that specifically measure BBB permeability differences between age, source, and culture matched male and female BMECs are needed.

A Master's thesis written by Dakota Kamm addresses sex differences in BBB permeability in the SAMP8 mouse model of accelerated aging.^92^ Kamm found female mice to have increased mRNA expression of claudin 1, 5, and 12, occludin, junction adhesion molecule A (JAMA), ZO-1, major facilitator superfamily domain containing 2, and brain-derived neurotrophic factor compared to their male counterparts.^92^ This study suggests sex-specific differences in tight junction protein expression that could affect BBB function. Sex differences in BBB characteristics should be further studied *in vivo* in other rodent models of neurodegenerative disease and *in vitro* using human cell lines.

### B. Sex differences in the BBB shear stress response and vascular function

Sex-dependent shear stress responses may also influence neurodegenerative disease susceptibility. Cerebral vasodilation is essential for supplying glucose to metabolically active brain regions by increasing local blood flow.^93^ A human brachial artery study suggested that the endothelium of pre-menopausal women may be more sensitive to shear stress and, thereby, increase vasodilation in response to shear stress as compared to endothelium from similarly aged men and post-menopausal women.^94^ Gracilis muscle arterioles isolated from female rats displayed an increase in flow-mediated dilation compared to male rats, which decreased wall shear stress and shear stress-induced endothelial damage.^95^ Pre-menopausal women also have decreased arterial^96^ and capillary pressure^97^ compared to men. Although these measurements were not taken in the brain, they suggest that women may have increased vasodilation in response to shear stress in the cerebral vasculature.

Sex may also influence vasodilation through endothelial-derived hyperpolarization (EDH). In EDH, G-protein coupled receptor (GPCR) stimulation increases intracellular calcium and hyperpolarizes the cell membrane. This hyperpolarization is thought to be transmitted via gap junctions to smooth muscle cells, which dilate the blood vessels and, thereby, increase cerebral vascular perfusion. A recent EDH aging study suggested that male mice have decreased GPCR function compared to age-matched female mice, leading to a decreased EDH response.^98^ On the contrary, female rat cerebral artery studies show that EDH is increased following ovariectomy and then decreased with estrogen supplementation, indicating that estrogen may decrease the EDH response. Of note is that the EDH response is attenuated by NO,^99^ which increases with estrogen,^100^ and so more studies are needed to confirm direct relationships between EDH and female sex hormones.

### C. Sex differences in BBB metabolism

Sex-related differences in metabolic aging and glucose metabolism, particularly in the brain, are highly debated. A recent study by Goyal *et al.* used a machine learning algorithm trained on positron emission tomography (PET) imaging of male vs female brains to compare the chronological age with the calculated metabolic age. Specifically, ^18^F-fluoro-2-deoxy-D-glucose uptake was used to quantify the cerebral glucose metabolic rate, oxygen consumption, cerebral blood flow, and aerobic glycolysis.^101^ The authors found that female brains have a lower metabolic age compared to male brains;^101^ however, critics of these data suggest that the trends may only apply post-puberty or may be an artifact in the machine learning algorithm.^102,103^ Cerebral hypometabolism is a phenotypic risk for neurodegenerative disease.^104^ Voxel-based PET scan analysis showed region-specific sex differences in cerebral glucose metabolism, with female brains having a higher glucose metabolic rate in the hypothalamus and male brains having higher glucose metabolic rates in the right insula, middle temporal gyrus, and medial frontal lobe.^105^ Additionally, gene expression profiles of male and female aging mice hippocampi indicated that while brain metabolism decreases overall with the age, this decrease occurs at an earlier time in female than in male brains.^104^ The earlier decrease in glucose metabolism in female brains may be associated with declining estrogen receptor expression following the menopausal transition.

Although little *in vitro* research has investigated sexual dimorphisms in BMEC metabolism, studies in other endothelial cell lines demonstrate apparent metabolic differences between male and female cells. Lorenz *et al.* recently showed in human umbilical vein endothelial cells (HUVECs) procured from male-female twin sets that VEGF-stimulated male HUVECs had higher mitochondrial respiration and lower glycolysis:mitochondrial respiration ratios than VEGF-stimulated female HUVECs.^106^ Furthermore, female HUVECs had higher intracellular adenosine triphosphate (ATP) following serum starvation than male HUVECs.^106^ HUVEC studies also demonstrated a positive correlation between estradiol (E2) binding and phosphofructokinase-2/fructose-2, 6-bisphosphatase 3 (PFKFB3) upregulation, indicating a potential link between sex hormones and a rate-limiting glycolytic enzyme.^107^ In the first BMEC differentiation from iPSCs, GLUT1 expression was higher in the female IMR90–4 cell line than in the male DF19–9-11T line.^85^ However, statistical analysis was not performed at the expression levels. These preliminary data justify further examination of sexual dimorphisms in BMEC glucose metabolism since these differences may lead to downstream energetic and functional discrepancies between male and female cells.

## V. POTENTIAL MECHANISMS BY WHICH ESTROGEN ALTERS THE BBB

### A. Estrogen increases NO production

**FIG. 2.**
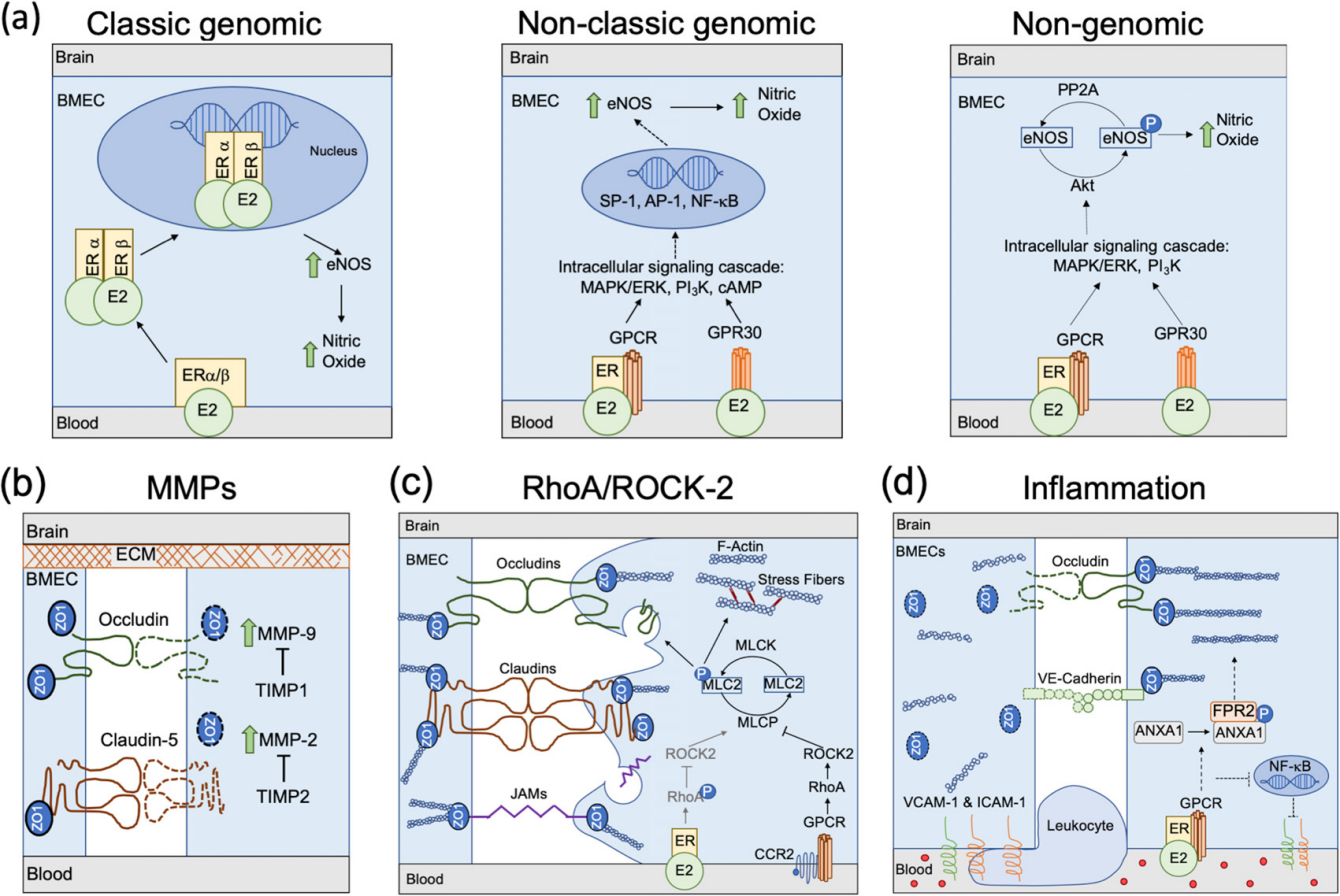
Mechanisms through which biological sex could affect BBB integrity. (a) Estrogen increases NO production. In the classic genomic pathway, estradiol (E2) binds to transmembrane estrogen receptors, which are then internalized and dimerize before binding to E2 response elements. The complex then regulates eNOS transcription and, thus, NO production. In the non-classic genomic pathway, E2 binds to either ER-associated GPCRs or GPR30, which triggers an intracellular signaling cascade including MAPK/ERK, PI_3_K, and cAMP. This then leads to increased eNOS transcription and NO production through co-factors such as SP-1, AP-1, and NF-κB. In the non-genomic pathway, E2 binding to ER-associated GPCRs or GPR30 activates Akt, which phosphorylates eNOS at Ser1177 and enables NO production. (b) Increased MMP-9 and MMP-2 or decreased TIMP1 and TIMP2 could lead to collagen IV degradation in the BBB extracellular matrix and break down claudin-5, occludin, and ZO1 to decrease BBB integrity. (c), Estrogen may inhibit the RhoA/ROCK2 pathway to maintain BBB integrity. Inflammatory cytokines bind to CCR2, which activates RhoA/ROCK2 to inhibit MLCP. This leads to tight junction protein internalization and degradation as well as actin stress fiber formation, which contracts the cell and pulls apart the BBB. E2 binding inhibits RhoA/ROCK2 to maintain the BBB. (d) In the presence of inflammatory cytokines, downstream GPCR signaling from E2 binding to ERs leads to ANXA1 phosphorylation, which stabilizes tight junction proteins and inhibits NF-κB to downregulate VCAM-1 and ICAM-1 expression on the plasma membrane. This then reduces the inflammatory response and leukocyte transmigration.

Endothelial nitric oxide synthase (eNOS) produces essential nitric oxide (NO), which locally dilates the cerebral vasculature to increase cerebral blood flow in response to metabolic need.^108^ eNOS, and thus NO production, can be increased via E2 binding to membrane-bound estrogen receptors (ERs).^100^ Both ER subtypes ER-α and ER-β are expressed in the cerebral vasculature,^109^ along with estrogen-binding G-protein coupled receptors (GPCRs), also called GPR30.^110^ Estrogen binding to ERs can initiate NO production through the classic genomic, non-classic genomic, or nongenomic pathway^111^ [Fig. 2(a)].

In the classic genomic pathway, E2 binding to ER-α or ER-β leads to E2-ER heterodimer internalization followed by intracellular ER-α and ER-β dimerization. The ER dimer then enters the cell nucleus, where it binds to E2 response elements (EREs). EREs regulate transcription of target genes, including eNOS.^112^ Intracellular ER dimers can also bind to EREs in mitochondrial DNA to decrease ROS production.^42^ Elevated ROS decreases NO bioavailability by reacting with NO to form reactive nitrogen species, which potentiates cell damage.^113^ In the non-classic genomic pathway, E2 binds to ERs, which initiates an intracellular signaling cascade involving mitogen-activated protein kinase (MAPK), extracellular signal-regulated kinase (ERK), phosphoinositide 3-kinase (PI_3_K), and cyclic adenosine monophosphate (cAMP).^112^ This cascade leads to indirect ER binding to DNA, mediated by co-factors such as SP-1, AP-1, and NF-κB, which, in turn, upregulates eNOS expression.^114,115^ The non-genomic pathway branches off the non-classic genomic pathway and does not require nuclear localization. After E2 binds to ERs, the intracellular MAPK/ERK/PI_3_K signaling cascade is initiated. Protein kinase B (Akt) is then activated to phosphorylate eNOS at Ser1177, leading to NO production.^116^

Ovariectomized mice demonstrate markedly increased NO production when given E2 supplements vs placebo.^117^ Cerebral microvessels from ovariectomized rats treated with E2 had a 17.4-fold increase in eNOS protein compared to rats treated with placebo. Postmenopausal women have a large drop in natural estrogen production that likely decreases NO production via the genomic and non-genomic ER pathways,^118^ while older men continue to metabolize testosterone to estrogen, thereby maintaining the ability to produce NO. Reduced estrogen-dependent NO production could be a contributing factor in decreased female cerebrovascular health post-menopause and the associated decrease in BBB function.

### B. MMP-9

The role of matrix metalloproteinases (MMPs) is debated in neurodegenerative disease research. MMPs are matrix remodeling proteins that degrade the extracellular matrix and tight junction proteins. There are 24 recorded human MMPs that are inhibited by tissue inhibitors of metalloproteinases (TIMPs).^119^ MMP-9 and MMP-2 are gelatinases and are associated with degradation of tight junction proteins and type IV collagen, a major endothelial basement membrane component.^120,121^ TIMP-1 and TIMP-2 are inhibitors for MMP-9 and MMP-2, respectively.^122,123^ In ischemic stroke studies, MMP-2 was involved in early tight junction and basement membrane protein degradation, while MMP-9 was implicated in long-term degradation.^121^ MMP-9 is essential for matrix remodeling in angiogenesis, which is neuroprotective; however, MMP-9 dysregulation could indicate disease pathology.^124^ Active MMP-2 and MMP-9 released from T cells, monocytes, and dendritic cells can also open the BBB, allowing leukocyte infiltration into the brain and contributing to the neuroinflammation associated with many neurodegenerative diseases [Fig. 2(b)].^119^

In studies of pulmonary tuberculosis and rheumatoid arthritis, serum collected from men had higher circulating MMP-9 compared to serum collected from women,^125,126^ which supports potential increased tight junction and basement membrane degradation and decreased BBB integrity in men. Further, peripheral blood mononuclear cells (PBMCs) collected from pregnant women treated with estriol (E3, an estrogen) significantly reduced MMP-9 production, indicating a probable role of estrogen in MMP-9 regulation.^127^ Conversely, E2 treatment increased active MMP-2 and MMP-9 in SH-SY5Y neuroblastoma cells, which model neurodegenerative diseases. These MMPs are hypothesized to increase degradation of amyloid-beta plaques associated with AD, thereby acting as neuroprotective proteins.^128^ Additional research is required to elucidate the mechanisms through which estrogen affects MMP-2 and MMP-9 activity and to determine if their activity perpetuates BBB breakdown and neurodegeneration or protects against it.

### C. RhoA/ROCK-2 Pathway

The RhoA/Rho-kinase-2 (ROCK-2) pathway, which can decrease BBB integrity through cytoskeletal remodeling, is mediated by estrogen^129^ [Fig. 2(c)]. During inflammation, circulating cytokines bind to C-C chemokine receptor type 2 (CCR2) to activate GPCRs. The resultant intracellular signaling cascade activates RhoA and ROCK-2, which, in turn, inhibit myosin light chain protease (MLCP). Without MLCP, myosin light chain 2 (MLC2) remains phosphorylated, leading to F-actin stress fiber formation and consequentially endothelial cell contraction.^130,131^ ROCK-2 activation additionally disconnects ZO proteins from actin and tight junction proteins, disbanding tight junctions and initiating tight junction protein endocytosis.^129^

Sex hormones affect the RhoA/ROCK-2 pathway although the results are controversial. E2 treatment inhibited the RhoA/ROCK-2 pathway in vascular smooth muscle cells in a time- and concentration-dependent manner. RhoA Ser188 phosphorylation blocked ROCK-2 activation and enabled MLCP to dephosphorylate MLC2.^72^ E2 replacement in ovariectomized PD mouse models decreased dopaminergic neuron death through RhoA/ROCK-2 pathway inhibition.^132^ In HUVECs, however, E2 treatment increased stress fiber formation and enhanced RhoA/ROCK-2 pathway activity.^131^ Future studies of the effects of estrogen on RhoA/ROCK-2 activity in the BBB are necessary to clarify the role of this pathway.

### D. Estrogen and Inflammation

Estrogen reduces BBB inflammation through annexin A1 (ANXA1), intercellular adhesion molecule 1 (ICAM-1),^19^ and vascular cell adhesion molecule-1 (VCAM-1). ANXA1 is a glucocorticoid anti-inflammatory messenger implicated in regulating BBB permeability.^133^ Maggioli *et al.* showed that estrogen binding to ERs associated with GPR30 promotes ANXA1 phosphorylation, which, in turn, binds to formyl peptide receptor 2 (FPR2) and stabilizes BBB tight junctions via actin reorganization^19,134^ [Fig. 2(d)]. Leukocytes adhere to the endothelium through ICAM-1 and VCAM-1. Leukocyte binding initiates cytoskeletal reorganization that pulls apart tight junctions, allowing activated leukocyte extravasation into the brain.^135,136^ Estrogen binding to GPR30 and subsequent ANXA1 phosphorylation downregulates ICAM-1 expression.^134^ Similarly, estrogen inhibits expression of NF-κB, a transcription factor responsible for both VCAM-1 and ICAM-1 production.^57^

Numerous *in vitro* and *in vivo* models have demonstrated how estrogen reduces inflammation through ANXA1 and cellular adhesion molecules. ANXA1^-/-^ mice challenged with lipopolysaccharide had decreased VE-cadherin and occludin expression and increased paracellular BBB permeability compared to healthy controls.^133^ Human aortic endothelial cells (HAECs) treated with tumor necrosis factor α (TNF-α) to induce inflammation reduced vascular cell adhesion molecule 1 (VCAM-1) and ICAM-1 following E2 treatment.^57^ Although these studies demonstrate protective estrogen effects on the BBB, neurodegenerative diseases associated with inflammation such as MS and AD disproportionately affect women. Thus, further research into the interactions among estrogen, inflammation, and the BBB is needed.

## V. CURRENT *IN VITRO* BBB MODELING TECHNIQUES

**FIG. 3.**
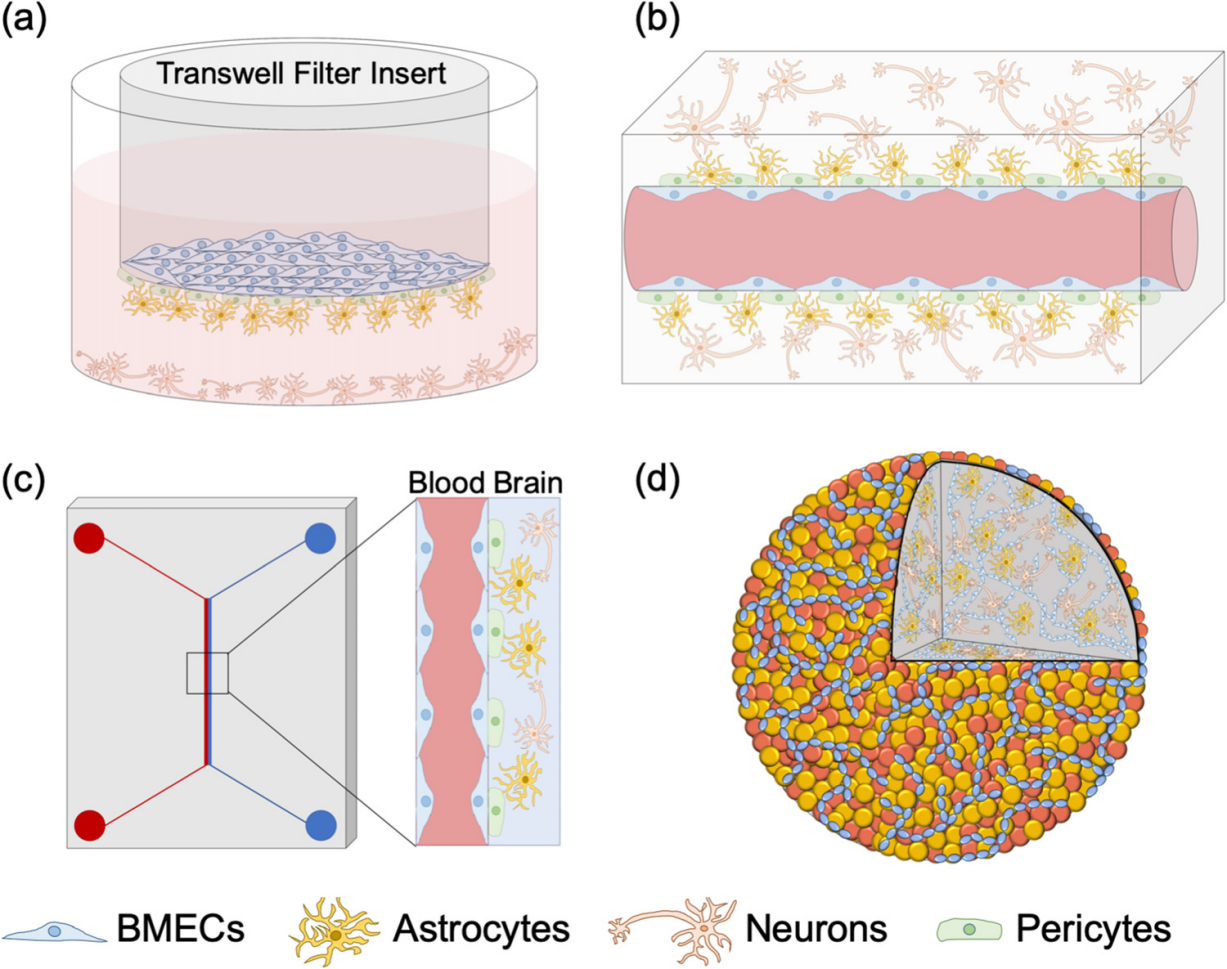
Current 3D *in vitro* models of a functional BBB. (a) Transwell filter models include a BMEC monolayer on top of the semi-permeable membrane, pericytes on the bottom of the semi-permeable membrane, and pericytes, astrocytes, and neurons below the filter. (b) Hydrogel models incorporate BMEC lining a hollow channel, and pericytes, astrocytes, and neurons dispersed in the surrounding hydrogel. (c) Microfluidic BBB models feature BMECs lining the blood compartment and pericytes, astrocytes, and neurons in the brain channel. (d) Organoid brain models include self-organized capillary networks representing the BBB, surrounded by neurons and astrocytes.

Four main strategies have been used to recapitulate the BBB structure and function *in vitro*: Transwell filters, hydrogel scaffolds, microfluidics, and organoids (Fig. 3). Importantly, all models incorporated BMECs, pericytes, astrocytes, and neurons. Each modeling technique is best suited for a different research objective. For example, the brain structure is best recapitulated with hydrogel scaffolds and organoids, while Transwell filters and microfluidics enable quantitative barrier strength measurements. In this section, we discuss in greater detail the design, advantages, and disadvantages of each model.

### A. Transwell filter BBB models

The Transwell filter BBB model [Fig. 3(a)] has two compartments separated by a semi-permeable membrane, which divides the vascular compartment from the brain compartment. Stone *et al.* recently devised a Transwell BBB model composed entirely of primary human cells in which astrocytes and pericytes were first seeded on the basal side and then a BMEC monolayer was seeded on the apical side.^137^ Finally, a plastic coverslip seeded with neurons was placed in the bottom of the well to produce a functional BBB.^137^ While there are variations in how the BBB is fabricated, the basic structure is conserved among Transwell filter BBB models.^138–141^ The main benefits of the Transwell culture system lie in its simple design and convenience for the trans-endothelial resistance (TEER) measurement as an assessment of *in vitro* BBB barrier strength.^89,137,141–143^ However, the Transwell filter pores limit interactions among cell types, and the basic Transwell system cannot incorporate mechanical stimuli (e.g., blood flow and substrate stiffness) without significant modification.

### B. Hydrogel BBB models

Hydrogel BBB models [Fig. 3(b)] are usually composed of a BMEC-lined hollow channel surrounded by a hydrogel that may include pericytes, astrocytes, and neurons. Most commonly, the hydrogel models are formed from a combination of collagen, hyaluronic acid, and gelatin to mimic brain stiffness (∼0.4–1.4 kPa).^91,144–148^ The hydrogel is crosslinked around a needle, and the needle is removed to form a straight channel. The channel is then lined with BMECs,^147^ either before or after the pericytes, and other brain cells are incorporated.^149^ Alternatively, BMECs, pericytes, and astrocytes can be homogenously mixed in a hydrogel, where they self-assemble to produce an *in vitro* BBB model.^150^ Hydrogel BBB model benefits include the ability to apply physiologically relevant shear stress to the BMEC-lined channel and tune the hydrogel stiffness. However, a constraint of hydrogel models is that the smallest channel diameter (∼25–35 *μ*m in self-assembled networks^151,152^) remains significantly larger than the 4–8 *μ*m inner brain capillary diameter.^153^ Hydrogel models also complicate genomic or proteomic measurements because the hydrogel needs to be degraded to extract and analyze the encapsulated cells.

### C. Microfluidic BBB models

Microfluidic BBB devices have channels constituting the “blood” and “brain” sides of the BBB [Fig. 3(c)] and are described in detail in reviews in the studies by Jiang *et al.*^154^ and Oddo *et al.*^155^ The “blood” channel is lined with BMECs, and the “brain” channel consists of the supporting cell types (pericytes, astrocytes, and neurons). In one microfluidic model, two polydimethylsiloxane (PDMS) channels were bonded together with a semi-permeable membrane separating them to form an apical and a basal channel.^156^ Pericytes and astrocytes were then seeded into the apical channel and BMECs into the basal channel.^156^ Microfluidic BBB models enable studies of shear stress effects on the BBB and are often used to study neuroinflammation and BBB barrier strength, sometimes integrating electrodes above and below the BMEC layer to measure TEER in real time.^157,158^ Microfabrication techniques such as two‐photon lithography have allowed 10 *μ*m channel fabrication in a microfluidic model, producing BBB structures close to the size of a brain capillary.^159^ However, microfluidic models limit protein, RNA, and DNA expression assays due to the low cell number that can be cultured in the microchannels.

### D. Organoid BBB models

Organoid BBB models take advantage of self-assembly to create cell spheroids with a 3D structure more similar to the brain, with the goal of producing a functional “mini-brain” for BBB research [Fig. 3(d)]. The details of brain organoid models are further discussed in a review in the study by Qian *et al.*^160^ Brain organoids can be produced by culturing cells in a low-attachment U-bottom 96-well plate,^161^ in agarose microwells,^162^ or in hanging drops.^163^ One BBB organoid model produced using the hanging-drop method self-assembled with neurons and astrocytes in the core, while BMECs and pericytes formed an outer shell.^163^ Current effort to advance BBB organoid models focuses on patterning perfusable vascular networks via bioprinting, sacrificial networks, stereolithography, direct 2-photon fabrication, subtractive fabrication, or inducing spontaneous vascularization.^164^ BBB organoids may best recapitulate 3D cortical brain structures and cell-cell interactions. However, organoid vessels cannot be perfused, which makes assaying BBB permeability a challenge. Because of oxygen diffusion limitations,^1^ BBB organoid models must remain small and are, therefore, limited in assay compatibility due to the low cell number in each organoid. Additionally, cell analysis within brain organoids is challenging because the cell types are not readily separated.

### E. BMECs for BBB modeling

Just as each BBB model has advantages and disadvantages, no single BMEC source is perfect for *in vitro* BBB models. Human primary BMEC cultures can be isolated postmortem and are likely the most physiologically relevant cells for BBB modeling; however, sources are limited, and they are best used at low passage as the cells have limited proliferative capacity and extended culture leads to dedifferentiation.^165^ Immortalized human BMEC lines were developed through lentiviral transfection of telomerase reverse transcriptase (hTERT) and the SV40 large T antigen into primary human BMECs.^166^ These cells express tight junction proteins, form capillary-like tubes in matrix, and can be maintained for at least 35 passages without dedifferentiating. However, these cells have suboptimal TEER values and may not respond to flow.^166^ Thus, while immortalized human BMECs are readily available and stable, experiments should be validated in primary cells *in vitro* and in animals or humans *in vivo*. iPSC-derived BMECs have robust barrier properties;^167^ however, recent RNA sequencing suggests these cells to have an underlying epithelial signature.^168–170^ iPSC-BMECs are easier to obtain and culture than primary BMECs, and iPSCs can also be differentiated into pericytes, astrocytes, and neurons to create a fully sex-specific model. However, variability in the cell source, as well as reprogramming and differentiating techniques, introduces confounding variables that may make it difficult to parse out cell sex effects.

## VI. SUMMARY AND FUTURE OUTLOOK

Many studies fail to address sex discrepancies in the BBB and its impact on neurodegenerative disease development and progression. Based on the aforementioned differences in barrier strength, shear stress response, and metabolism, both cell sex and sex hormone exposure need to be considered in the BBB. *In vitro* BBB models are particularly powerful in determining how both sex and the microenvironment affect BBB function. Male and female BMECs with and without sex hormone stimulation can be incorporated in Transwell filter models to study barrier strength, in hydrogel models to study matrix stiffness, in microfluidic models to study shear stress, and in organoid models to study drug neurotoxicity.

The biochemical differences between men and women go beyond estrogen. Other sex hormones such as progesterone and testosterone may also influence BBB integrity. Significant sex differences were further found in 61 of 71 circulating cardiovascular disease biomarkers, including ceruloplasmin, which exists in higher levels in women than men and is decreased in association with Parkinson's disease.^171,172^ These differences in circulating factors may then affect endothelial cells and the BBB. Indeed, serum from sedentary men induced higher ROS levels compared to serum from sedentary women when added to HUVECs,^173^ despite no differences in the circulating estradiol concentration. Human serum from men and women could be applied to cells of the same and opposite sex to observe the influence of other sex-related biochemical differences on BBB properties.

Differences between male and female gene expression are also prevalent. A comprehensive study by Olivia *et al.* suggests that 37% of all genes display sexual dimorphisms in at least one tissue.^174^ Additionally, genes that occasionally escape X-chromosome inactivation, such as TIMP1^175^ and O-linked N-acetylglucosamine transferase (OGT),^176^ decrease BBB breakdown^177^ and metabolic processes related to neurodegeneration,^178^ respectively. These intrinsic sex-related gene expression differences have also been found in endothelial cells. Indeed, a study of human endothelial genes at birth and in adults has shown that between 14% and 25% of the endothelial cell transcriptome is influenced by sex.^179^

Finally, men and women also have essential differences in cardiovascular biomechanics. Women have lower arterial and capillary blood pressures, smaller arterial diameters, and increased arterial stiffness all with similar cardiac output when compared to men.^180^ Additionally, brain stiffness declines with age, and one study using magnetic resonance elastography determined that adult women have stiffer temporal and occipital lobes than age-matched men.^181^ The tunable stiffness of the hydrogel platforms, as well as the ability to modify flow properties in hydrogel and microfluidics models, enables the study of age-, disease- and sex-related vascular stiffness and shear stress.

The influence of sex in BBB disruption and neurodegenerative diseases is understudied. While animal models allow the *in vivo* study of sexual dimorphisms in neurodegenerative diseases, the results from animal models often fail to translate to the human condition. Advances in BBB modeling enable us to study hormonal and non-hormonal BBB sex differences in 3D, physiologically relevant human models. Furthermore, *in vitro* models can be produced using iPSCs reprogrammed from patients with the disease of interest to study the effects of sex hormones, as well as to investigate interactions of the cells with their mechanical environment. Integrated *in vitro* and *in vivo* studies of sex differences in the BBB will improve our understanding of the complex relationships between sex and BBB function and could enhance personalized medicine for neurodegenerative disease.

## AUTHORS' CONTRIBUTIONS

C.M.W. and A.M.C. conceived and wrote this manuscript.

## Data Availability

Data sharing is not applicable to this article as no new data were created or analyzed in this study.
